# Pneumomediastinum and Subcutaneous Emphysema in an Adult Male From Nepal Infected With COVID-19

**DOI:** 10.7759/cureus.16306

**Published:** 2021-07-10

**Authors:** Satyasuna Kafle, Elina Shrestha, Nisheem Pokharel, Pravash Budhathoki, Dhan B Shrestha, Timothy Vittorio

**Affiliations:** 1 Department of Intensive Care Unit, Bhaktapur Hospital, Bhaktapur, NPL; 2 Department of Internal Medicine, BronxCare Health System, Bronx, USA; 3 Department of Emergency Medicine, Kist Medical College, Kathmandu, NPL; 4 Department of Internal Medicine, Mount Sinai Hospital, Chicago, USA; 5 Cardiology, BronxCare Health System, Bronx, USA

**Keywords:** covid-19, mediastinal emphysema, nepal, pneumothorax, respiratory distress syndrome

## Abstract

Pneumomediastinum and subcutaneous emphysema have been reported in COVID-19 around the world except for Nepal. We report a case of a 44-year-old male infected with COVID-19 who developed pneumomediastinum and subcutaneous emphysema during his eighth day of intubation at the hospital. He was managed with remdesivir, antibiotics, mechanical ventilation, steroid, and heparin following which he recovered well. Barotrauma-related complications are common in COVID-19 and our case highlights the importance of conservative management for such complications and the rarity of such conditions in Nepal.

## Introduction

The novel severe acute respiratory syndrome coronavirus 2 (SARS-CoV-2) was first reported in Wuhan, China in December 2019 and has evolved as a global coronavirus disease-2019 (COVID-19) pandemic [1]. As of June 12, 2021, there have been 176 million confirmed cases of COVID-19 reported worldwide with 3.8 million deaths [2]. The most prevalent manifestations of COVID-19 are fever, cough, and dyspnea [3,4]. severe acute respiratory syndrome coronavirus (SARS-CoV-2) has been reported to be associated with complications such as arrhythmia, acute respiratory distress syndrome (ARDS), shock, thromboembolic complications, myocardial injury, liver injury, renal injury, and multiple organ dysfunction syndromes (MODS) [5-7]. Computed tomography (CT) scan, which had primarily played a pivotal role as a tool for diagnosis with findings such as ground-glass opacities, opacities with rounded morphology, and crazy-paving pattern, has turned out to be useful to identify associated complications such as pneumothorax, pneumomediastinum, subcutaneous emphysema, and pneumatoceles which are emerging complications seen lately but are rarely reported [8,9]. Chen et al. reported that 1% of patients with COVID-19 developed pneumothorax [4].

Here we present a case of pneumomediastinum and subcutaneous emphysema in a COVID-19 patient which is rare in Nepal and discuss its clinical course and management. The case report is reported as per the Surgical CAse REport (SCARE) guidelines [10].

## Case presentation

A 44-year-old healthy male with no significant past medical history presented to the emergency department with fever and cough for five days and worsening shortness of breath for two days. He had no palpitation, syncope, altered sensorium, and altered bowel habits. On the initial assessment, the patient had an oxygen saturation (SpO2) of 58% in room air, tachycardia with a pulse rate of 101 beats per minute, tachypnea (respiratory rate of 26 breaths per minute), fever (100.1 F), and blood pressure (BP) of 140/90 mmHg. The patient was placed on a non-rebreather mask with 15 liters of oxygen. His lab parameters at admission are presented in (Table 1).

**Table 1:** Lab Parameters of the patient at admission mg/dl: milligram per decilitre; mEq/l: milliequivalent per liter; mm3: cubic meter

Total Leukocyte Count (TLC)	7,600/ mm3
Platelets	3,74,000/ mm3
C-Reactive Protein (CRP)	1.12 mg/dl
Sodium	144 mEq/l
Potassium	4.5 mEq/l
Real Time Polymerase Chain Reaction for COVID-19 (RT-PCR)	Positive
Urea	32 mg/dl
Creatinine	1.2 mg/dl

**Figure 1:**
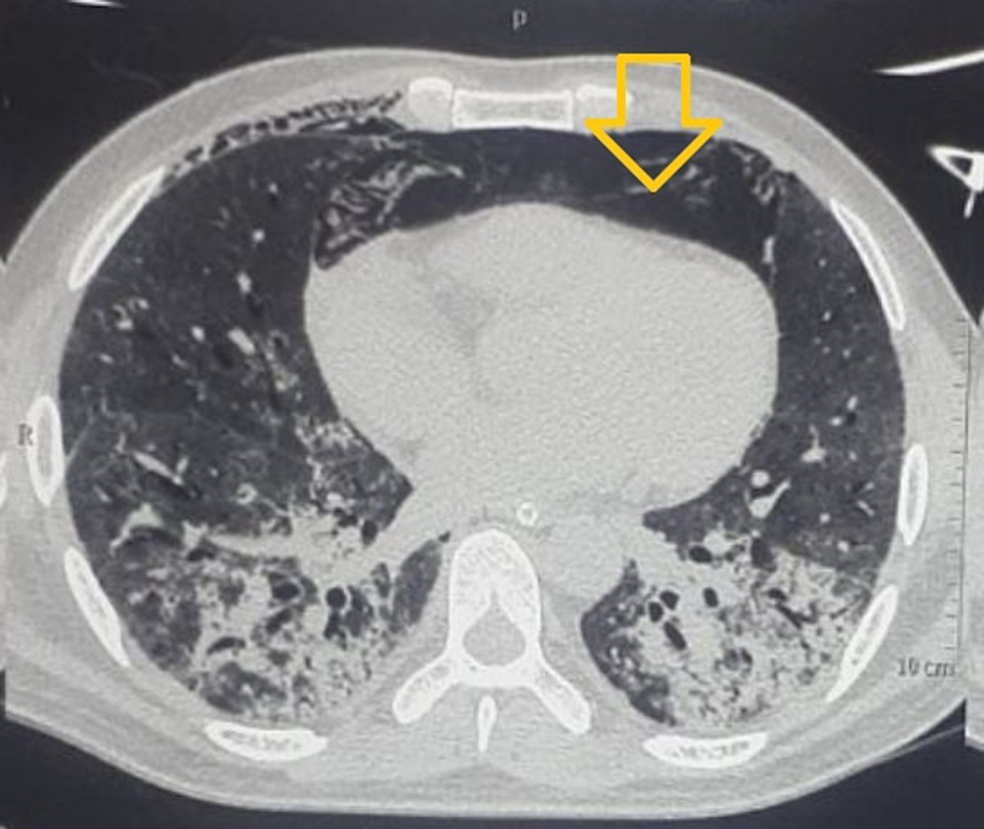
Transverse slice of CT image from mid-thoracic level showing pneumomediastinum (shown by orange arrow)

**Figure 2:**
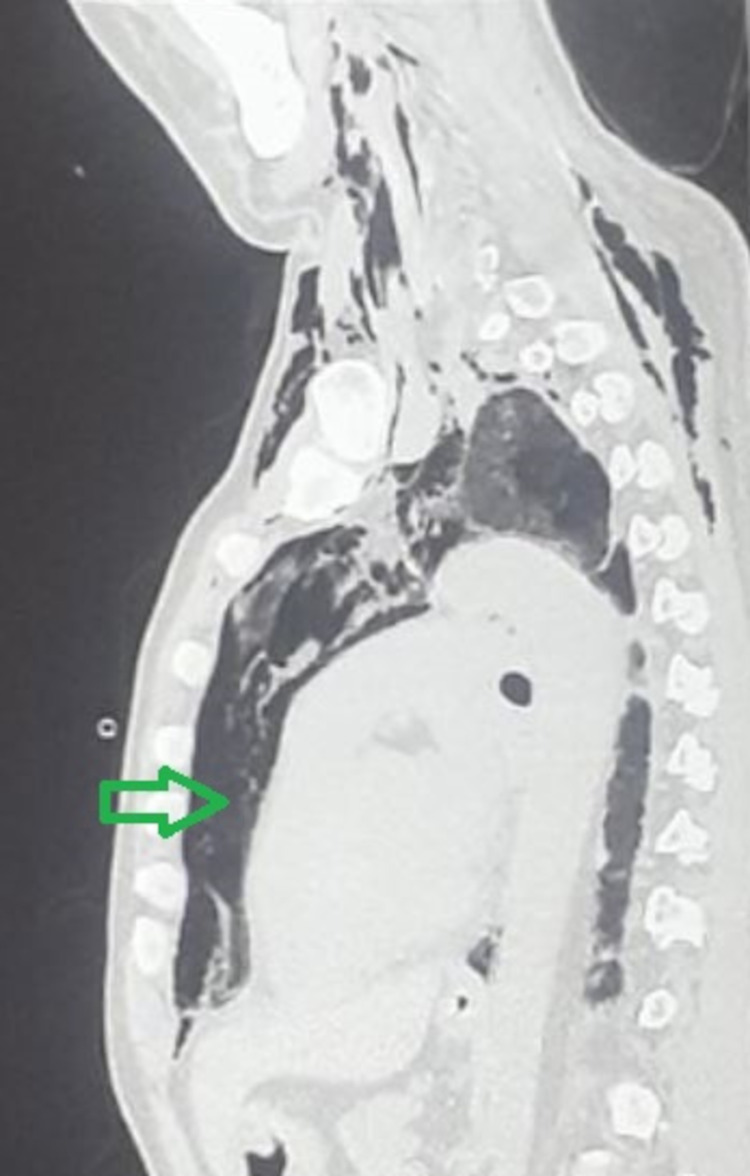
Left Parasagittal slice of CT image showing finding of pneumomediastinum (shown by a green arrow)

**Figure 3:**
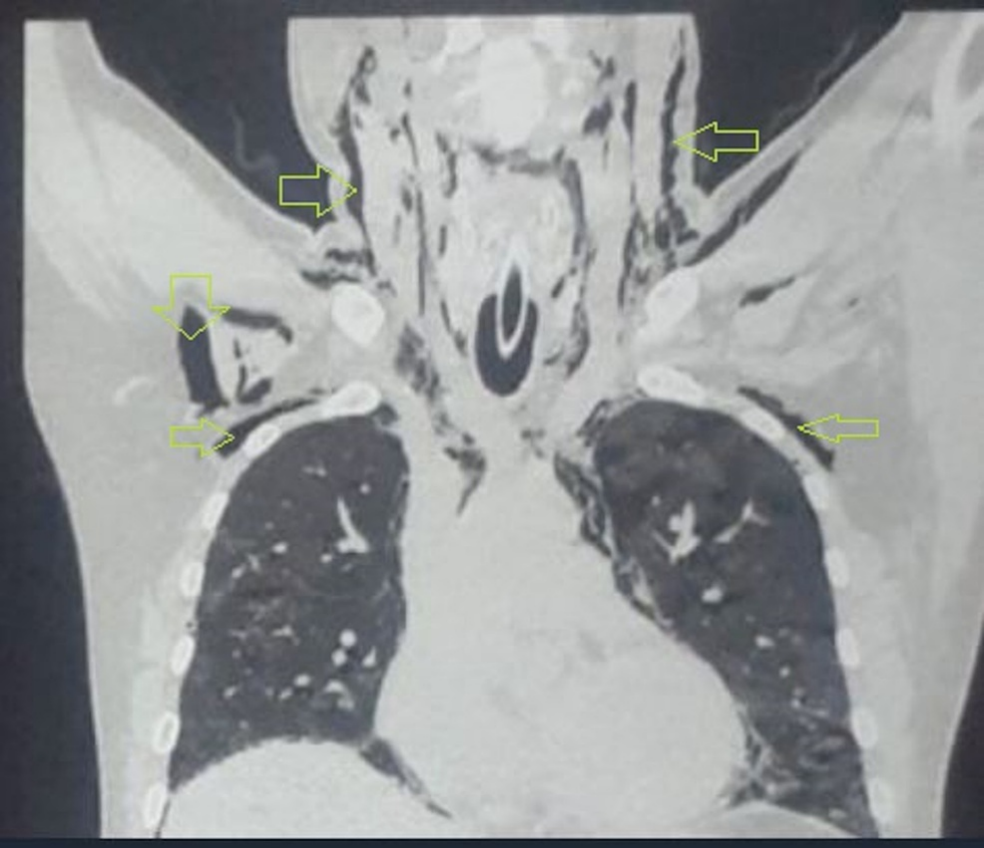
Coronal view of CT showing subcutaneous emphysema (shown by yellow arrows)

The patient was admitted to the high dependency unit (HDU). His initial treatment included antibiotics (tazobactam+piperacillin and azithromycin), remdesivir, dexamethasone, enoxaparin, vitamins (vitamin C, D, B-complex), antihistamines, and antipyretics. But, due to an increment in his oxygen requirement, he was kept on intermittent bimanual positive airway pressure (BiPAP) and was shifted to the intensive care unit (ICU) on day two of admission. On day seven in ICU, the patient’s condition further deteriorated, the oxygen saturation declined, and he was kept in a non-invasive ventilator continuous positive airway pressure (NIV CPAP) with FiO2 100%, positive end-expiratory pressure (PEEP) of 10 cm of H20 (water), and pressure support of 5 cm of H20 (water). However, the patient further deteriorated, then he was intubated the following day and was placed on invasive mechanical ventilation. His total leukocyte count (TLC) was gradually increasing but fungal yeast cells were seen in sputum culture. On day 12 of admission, his antibiotics were upgraded to meropenem and teicoplanin, and fluconazole was also added. On the eighth day of intubation (18th day of admission), subcutaneous emphysema was noticed at the upper chest, neck, and cheeks. So, a high-resolution computed tomography-chest (HRCT-Chest) was done which showed subcutaneous emphysema in bilateral neck regions, anterior and bilateral chest walls, and bilateral axillary regions with a very high CT severity index of 24/25 (Figures 1-3). Also, pneumomediastinum was noted in the same HRCT.

The patient was managed conservatively, and invasive mechanical ventilation was continued. Subcutaneous emphysema resolved gradually, and the patient became symptomatically better. His condition gradually improved, and after 24 days of admission and 16 days of mechanical ventilation, he was extubated. After 28 days of ICU stay, he was shifted to HDU and then to the medical ward. He is currently stable on nasal prongs with 1-2 liters of oxygen support. 

## Discussion

There are several studies reporting the spontaneous pneumothorax, pneumomediastinum, or subcutaneous emphysema in COVID-19 during its various disease stages; as presenting symptoms for diagnosis of COVID-19, during the hospital course, and even after recovery of the COVID-19 [11-17]. Our report is the first in Nepal to report pneumomediastinum and subcutaneous emphysema in a patient diagnosed with COVID-19 in Nepal seen during the hospital course. A prospective study was conducted on 75 patients in Hongkong by Peiris et al., who found the incidence of spontaneous pneumomediastinum to be 12 % [18]. Our patient developed two of the complications, pneumomediastinum, and subcutaneous emphysema during the hospital course of the disease. Pneumothorax in patients with COVID-19 has been proposed to be related to cystic and fibrotic changes leading to alveolar tears in the lung parenchyma. In addition, the increase in intrathoracic pressure resulting from prolonged coughing and/or mechanical ventilation can also be related to the cause [19]. A retrospective analysis done at a tertiary care center in Philadelphia showed that 0.66% of COVID-19 positive patients developed spontaneous pneumothorax, and the risk increased among those with baseline ground-glass opacities, and those who required mechanical ventilation [19]. Our patient underwent invasive mechanical ventilation for oxygenation and later developed complications on day eight of intubation like pneumomediastinum and subcutaneous emphysema. We believe the COVID-19 positive state put him more at risk for developing these complications, however, our patient did not develop pneumothorax.

Lemmers et al. performed a retrospective study on two cohorts of patients with 169 patients, CoV-ARDS, and 163, non-CoV-ARDS. They found that the occurrence of pneumomediastinum and subcutaneous emphysema was rare in non-CoV-ARDS as compared to CoV-ARDS even if the ventilatory approach was the same. Thus, they attributed the cause to the increased lung frailty state of CoV-ARDS rather than the barotrauma used to treat respiratory distress [20]. 

In a retrospective cohort study on COVID-19 patients, McGuinness et al. found the incidence of pneumothorax, pneumomediastinum, pneumopericardium, subcutaneous emphysema to be 15% in patients with COVID-19 requiring invasive mechanical ventilation, excluding the barotrauma related to line or surgical procedure [21]. There have been mixed findings reported on the studies regarding the poor prognosis of the disease due to these complications. The studies regarding such complications leading to mortality or poor prognosis have been inconclusive. Retrospective analysis of 92 deceased patients with COVID-19 was done by Yang et al. from Jan 6, 2020, to Feb 25, 2020, in Renmin Hospital of Wuhan University in which they found one case of death due to pneumothorax [5]. Martinelli et al. analyzed multicentre retrospective cases in the UK with patients diagnosed with COVID-19 and having pneumothorax or pneumomediastinum from March to June 2020, with follow-up post pneumothorax. The incidence of pneumothorax was 60 out of 71 total cases reviewed; six out of the 60 with pneumomediastinum, and 11 out of 71 with pneumomediastinum alone. They found that the 28-day survival was not significantly different, so pneumothorax could not be an independent marker for poor prognosis [22]. Akdogan et al. reported a case series of five mechanically ventilated patients with COVID-19 infection who developed pneumothorax. Three out of five patients died, suggesting pneumothorax to be a potentially fatal complication of the COVID-19 [23]. 

The presence of spontaneous pneumomediastinum in COVID-19 was associated with severe clinical course requiring aggressive management in a retrospective analysis by Loffi et al. in a tertiary care center in Northern Italy which was the first European area to be hit by COVID-19 [24]. Wang et al. performed a retrospective analysis of 248 COVID-19 patients and found the incidence of pneumothorax to be 24% in those with mechanical ventilation, the overall incidence was 2.01% and incidence in patients with ARDS was 10%. And the mortality was as high as 80%; four out of the five [25]. A multicentre case-control study in Spain by Miro et al. found that the incidence of spontaneous pneumothorax was low, but was higher in COVID-19 as compared to non-COVID patients. And, they also concluded that COVID-19 patients with spontaneous pneumothorax had worse outcomes when compared to non-COVID-19 patients or COVID-19 patients without pneumothorax [26]. It can be argued that the sample size of the studies and the co-morbidities could have biased the varied results. Thus, large studies are needed to establish a causal relationship with confidence. Fortunately, our patient recovered well. 

A case series of pneumomediastinum post-intubation on COVID-19 patients was studied by Wali et al. [27], in which two out of five of them died. They recommend the use of bilateral chest and subcutaneous drains in severe cases that requires decompression [27]. Volpi et al. studied the management of pneumomediastinum in three case reports. They emphasize conservative treatment with gradual resorption of air from the tissues with special attentiveness to decompensation [28]. Our patient improved with supportive management for his diseased state rather than specific therapy targeted for the complications of pneumomediastinum and subcutaneous emphysema. Further researches are required for guidelines in the management as definite treatment has not been established yet. We recommend the health care workers be vigilant of complications like subcutaneous emphysema and pneumomediastinum, and use their knowledge in the management of the patients while fighting this pandemic. 

## Conclusions

The complications of COVID-19 like pneumomediastinum and subcutaneous emphysema have been reported several times through various case reports. We share our case as an addition to the published cases to emphasize its rarity in Nepal and the importance to rule out pneumothorax or pneumomediastinum when a patient with COVID-19 has an acute deterioration in respiratory function.

## References

[1] Zhu H, Wei L, Niu P (2020). The novel coronavirus outbreak in Wuhan, China. Glob Health Res Policy.

[2] (2021). COVID Live Update: 176,019,737 Cases and 3,799,792 Deaths from the Coronavirus - Worldometer. https://www.worldometers.info/coronavirus/.

[3] Rodriguez-Morales AJ, Cardona-Ospina JA, Gutiérrez-Ocampo E (2020). Clinical, laboratory and imaging features of COVID-19: a systematic review and meta-analysis. Travel Med Infect Dis.

[4] Chen N, Zhou M, Dong X (2020). Epidemiological and clinical characteristics of 99 cases of 2019 novel coronavirus pneumonia in Wuhan, China: a descriptive study. Lancet.

[5] Yang F, Shi S, Zhu J, Shi J, Dai K, Chen X (2020). Analysis of 92 deceased patients with COVID-19. J Med Virol.

[6] Klok FA, Kruip MJ, van der Meer NJ (2020). Incidence of thrombotic complications in critically ill ICU patients with COVID-19. Thromb Res.

[7] Wang D, Hu B, Hu C (2020). Clinical characteristics of 138 hospitalized patients with 2019 novel coronavirus-infected pneumonia in Wuhan, China. JAMA.

[8] Ai T, Yang Z, Hou H (2020). Correlation of chest CT and RT-PCR testing for coronavirus disease 2019 (COVID-19) in China: a report of 1014 cases. Radiology.

[9] Chung M, Bernheim A, Mei X (2020). CT Imaging features of 2019 novel coronavirus (2019-nCoV). Radiology.

[10] Agha RA, Franchi T, Sohrabi C, Mathew G, Kerwan A (2020). The SCARE 2020 guideline: updating consensus surgical case report (SCARE) guidelines. Int J Surg.

[11] Elhakim TS, Abdul HS, Pelaez Romero C, Rodriguez-Fuentes Y (2020). Spontaneous pneumomediastinum, pneumothorax and subcutaneous emphysema in COVID-19 pneumonia: a rare case and literature review. BMJ Case Rep.

[12] Abushahin A, Degliuomini J, Aronow WS, Newman T (2020). A case of spontaneous pneumothorax 21 days after diagnosis of coronavirus disease 2019 (COVID-19) pneumonia. Am J Case Rep.

[13] Wang W, Gao R, Zheng Y, Jiang L (2020). COVID-19 with spontaneous pneumothorax, pneumomediastinum and subcutaneous emphysema. J Travel Med.

[14] Ucpinar BA, Sahin C, Yanc U (2020). Spontaneous pneumothorax and subcutaneous emphysema in COVID-19 patient: case report. J Infect Public Health.

[15] Quincho-Lopez A, Quincho-Lopez DL, Hurtado-Medina FD (2020). Case report: pneumothorax and pneumomediastinum as uncommon complications of COVID-19 pneumonia-literature review. Am J Trop Med Hyg.

[16] Utomo SA, Notopuro F, Rosalina S (2021). Massive emphysema subcutis, pneumothorax, pneumomediastinum and pneumoperitoneum as uncommon complication of covid-19 pneumonia, a rare case. Radiol Case Rep.

[17] Shan S, Guangming L, Wei L, Xuedong Y (2020). Spontaneous pneumomediastinum, pneumothorax and subcutaneous emphysema in COVID-19: case report and literature review. Rev Inst Med Trop Sao Paulo.

[18] Peiris JS, Chu CM, Cheng VC (2003). Clinical progression and viral load in a community outbreak of coronavirus-associated SARS pneumonia: a prospective study. Lancet.

[19] Zantah M, Dominguez Castillo E, Townsend R, Dikengil F, Criner GJ (2020). Pneumothorax in COVID-19 disease- incidence and clinical characteristics. Respir Res.

[20] Lemmers DH, Abu Hilal M, Bnà C (2020). Pneumomediastinum and subcutaneous emphysema in COVID-19: barotrauma or lung frailty?. ERJ Open Res.

[21] McGuinness G, Zhan C, Rosenberg N (2020). Increased incidence of barotrauma in patients with COVID-19 on invasive mechanical ventilation. Radiology.

[22] Martinelli AW, Ingle T, Newman J (2020). COVID-19 and pneumothorax: a multicentre retrospective case series. Eur Respir J.

[23] Akdogan RE, Mohammed T, Syeda A, Jiwa N, Ibrahim O, Mutneja R (2021). Pneumothorax in mechanically ventilated patients with COVID-19 infection. Case Rep Crit Care.

[24] Loffi M, Regazzoni V, Sergio P (2020). Spontaneous pneumomediastinum in COVID-19 pneumonia. Monaldi Arch Chest Dis.

[25] Wang XH, Duan J, Han X (2021). High incidence and mortality of pneumothorax in critically Ill patients with COVID-19. Heart Lung.

[26] Miró Ò, Llorens P, Jiménez S (2021). Frequency, risk factors, clinical characteristics, and outcomes of spontaneous pneumothorax in patients with coronavirus disease 2019: a case-control, emergency medicine-based multicenter study. Chest.

[27] Wali A, Rizzo V, Bille A, Routledge T, Chambers AJ (2020). Pneumomediastinum following intubation in COVID-19 patients: a case series. Anaesthesia.

[28] Volpi S, Ali JM, Suleman A, Ahmed RN (2020). Pneumomediastinum in COVID-19 patients: a case series of a rare complication. Eur J Cardiothorac Surg.

